# A Decentralized Framework for Multi-Agent Robotic Systems

**DOI:** 10.3390/s18020417

**Published:** 2018-02-01

**Authors:** Andrés C. Jiménez, Vicente García-Díaz, Sandro Bolaños

**Affiliations:** 1 Department of Electronic Engineering, Los Libertadores Foundation University, Cr.16#63A-68 Bogotá, Colombia; 2 Department of Computer Sciences, University of Oviedo, Street San Francisco 1, 33003 Oviedo, Asturias, Spain; garciavicente@uniovi.es; 3 Department of Engineering, Distrital University Francisco José de Caldas, Cr.7#40B-53 Bogotá, Colombia; sbolanos@udistrital.edu.co

**Keywords:** communication, decentralization, distributed systems, multi-agent robotic systems

## Abstract

Over the past few years, decentralization of multi-agent robotic systems has become an important research area. These systems do not depend on a central control unit, which enables the control and assignment of distributed, asynchronous and robust tasks. However, in some cases, the network communication process between robotic agents is overlooked, and this creates a dependency for each agent to maintain a permanent link with nearby units to be able to fulfill its goals. This article describes a communication framework, where each agent in the system can leave the network or accept new connections, sending its information based on the transfer history of all nodes in the network. To this end, each agent needs to comply with four processes to participate in the system, plus a fifth process for data transfer to the nearest nodes that is based on Received Signal Strength Indicator (RSSI) and data history. To validate this framework, we use differential robotic agents and a monitoring agent to generate a topological map of an environment with the presence of obstacles.

## 1. Introduction

Single robots are made to solve problems over a unique domain, preventing the expansion of their use beyond its original purpose. The cost of their development usually is expensive and does not have strategies to solve failure problems by itself [1]. Furthermore, a single robotic agent needs complex algorithms to solve some navigation task, which implies a powerful unit to process all the information. In contrast, a Multi-Agent Robotic System (MARS) is characterized by its fault-tolerant strategies, where, if an agent of the system crashes, another can occupy its place. Another remarkable characteristic of MARS is the ability to adapt and process easily a complicated geographical environment. As each agent processes different places, they reduce the time to cover the scenario and the workload of the system compared by the time than a single unit can achieve [2,3].

It has been proven that a MARS is capable of performing search and rescue tasks of people in difficult to access environments [4], building monitoring and surveillance [5], sensor deployment and measurement in unknown environments in a way that is more efficient than using a single robotic agent, for map generation and capture [6,7]. For these tasks, MARS requires a distributed control structure for its individual agents, so each of them works autonomously (does not require a human operator to function). This is possible because agents cooperate with each other by sharing information in a network.

The distributed control network can be centralized or decentralized. In the centralized model, the system depends on a central unit, which requires all the information to make decisions. In a decentralized structure, each agent of the MARS processes its information locally [8,9]. Furno et al. [10] compare these two types of structures by using a system of three robotic submarine agents tasked with equipment transportation. While the centralized version of the system requires less time to correct errors in the actuators to update direction and speed, the decentralized system presents the advantages of being fault tolerant in the event of system failure of one robotic agent and having a smaller packet size in transmissions between agents, which is desirable given the bandwidth limitations of the communications channel. However, information exchange between the three units must be constant in both the centralized and decentralized models.

The fault tolerance in decentralized distributed control systems is leveraged in various areas. Schwager et al. [11] propose sensor deployment through a MARS where each agent shares information only with the nearest neighbor within a convex area. This means that this implementation does not take into account discontinuity in the environment, be that in the form of holes or other obstacles in the environment or work area. Cheng et al. [12] implements a robust system for survey and information capture through sensors in a predetermined area, keeping uninterrupted communication as a means to avoid errors in speed and direction for each mobile robot.

Another type of application is formations of mobile robots using roles and network topologies specific for network communication optimization. This is a decentralized multi-agent model because it only transmits information between neighboring nodes, but centralizes operations by depending on a leader within the formation or, in adaptive systems, by sharing a cost-reward function to modify the system [13,14,15]. Another salient characteristic of MARS is their capacity for being heterogeneous, as they can be comprised of different types of units. Tanner’s work [16] proves that MARS’s can be heterogeneous and decentralized by including Unmanned Aerial Vehicles (UAV) and Unmanned Ground Vehicles (UGV), pointing out that the resulting system is decentralized by not depending of a central unit, but it is dependent on permanent communication with a specific topology.

The issue of dependency on a specific topology has been investigated by Shang [17,18]; he established that mobile agents in a discrete environment can reach state consensus, i.e., exchange and sharing of information over all the agents performing random walks on a network, where the state of consensus is done when each random walk is an ergodic Markov chain. Furthermore, Shang shows that, if the agents are independent, they can be added to the network and share information if they occupy the same site with other agent simultaneously, but to achieve the synchronization of the system they only can be disconnected for a small time.

On the other hand, the requirement of a permanent communication between agents or with a central unit can be avoided by using ad hoc networks. Ming et al. [19] propose a MARS-based autonomous exploration system, proving that the agents in the system can be connected or disconnected from the network at any given moment, making it free from the requirement of having a permanent connection between agents. However, their work does not provide a study to avoid packet loss and the possibility of connecting with additional agents to complement the system.

This paper presents a framework for communication between multiple agents that eliminates the requirement of having a permanent link between robotic agents, based on the model presented in our previous works [20], improving the functionality by removing the time dependency to share the information between the agents, and enabling the connection of external units as backup, either for monitoring or to complete the assigned task in a decentralized manner by using ad hoc networks.

This framework is evaluated in a non-convex environment that includes discontinuities in the form of obstacles (walls), using differential mobile robotic agents to independently generate a topological map through odometry, which they share using the framework to complete the map in a decentralized way.

The present article is organized as follows. Section 2 presents the existing works that motivated the design of this framework. Section 3 describes each process of the framework and their implementation. Section 4 develops the experiment through the V-Rep simulator to model the robotic agents and the environment, using Python scripts to emulate the instructions used in the physical MARS agents. Finally, Section 5 presents the conclusions with consideration to future works.

## 2. Related Works

In Multi-Agent Robotic Systems, information distribution and processing are autonomous and have a high level of fault-tolerance, due to their modularity and distributed architecture [21,22]. System modularity allows the system to be robust because it can detect and easily replace agents or parts of them that are not working. Additionally, if the system needs to be partially updated, only the necessary agents need to be changed, reducing maintenance costs [23,24]. Being a distributed system results in the ability to perform multiple tasks simultaneously and asynchronously, without depending on a global control [25].

The approach to communication between agents is through a Wireless Sensor Network (WSN), as they are comprised of nodes with independent processing units, wireless communication modules, and sensors. The nodes in a WSN can be static or mobile, as is the case of robotic agents [26,27]. The use of this technology allowed to expand the applications of MARS. Garcia et al. [28] use WSNs to receive information from multiple agents to increase resource use efficiency in the administration of a freight airport. In this particular case, the robotic agents are static actuators that, although sharing information locally, depend on a single control system for information processing.

In emergency environments, WSNs enable MARS’s to interact within the environment by using this type of communications infrastructure, while enabling locating robotic agents through the use of Received Signal Strength Indicator (RSSI). This system is decentralized, as it only has one central unit for monitoring, but it requires WSN nodes to be pre-installed in the environment [29,30,31]. To remove this dependency on pre-installed sensors, techniques have been proposed to deploy MARS in tasks of Simultaneous Localization and Mapping (SLAM), in which a central unit is tasked to generate the map transmitted by the robotic agents [6,32,33,34].

Several decentralized control strategies have been proposed to eliminate the dependency on a central unit in MARSs, based on sharing information with the nearest neighbor in the system. For sensor deployment, Schwager et al. [11] assigned each agent in the MARS a sensor to be deployed in an area, using consensus algorithms to determine their final positions, and limiting the study to a convex environment—not in a real environment. In tasks of deployment, mapping, surveillance, and formation correction, control strategies haven proven system decentralization in non-convex environments but do not estimate interaction with other agents heterogeneous to the system, that is, they do not allow the incorporation of different agents [12,13,14,35].

Other approaches show the interaction with different types of robotic agents in heterogeneous systems comprised of UAV and UGV [16], in which decentralization was based on a control system with permanent communication between ground units using a unidirectional communication scheme between ground and aerial units. This scheme did not allow information exchange between the different groups of agents as a strategy to increase the level of fault tolerance.

To solve the problem of information exchange, previous works developed a decentralized communication model for MARS [20], in which each robotic agent starts with a list of all the nodes in the wireless network from the Service Set Identifier (SSID). For this reason, the agent cannot accept new connections after the system starts. Once the list is initialized, every agent begins their assigned navigation task individually, consisting in planning [36,37], localization [33,38], or mapping [7,39]. During the navigation phase, agents scan the network to see which other nodes are in range and select the node with the highest RSSI to establish a direct communication to transfer information. To avoid redundant information transmission, the data packet header includes the name of the agent and a key indicating a unique, transmission identifier that depends on the time of the last package sent. If the agent attempts to transfer a new package, but the current ID matches the last package sent in its transfer history, no information is sent. To avoid the loss of information, the model with the Transmission Control Protocol/Internet Protocol (TCP/IP) was implemented. If the system gets stuck trying to recover a package, the model includes a time-out flag to end the transmission.

The present article continues and improve the work of Jimenez et al. [20], proposing a decentralized framework for tasks that enables the connection of heterogeneous agents to the MARS without requiring a permanent connection to work. This is possible through the use of non-infrastructure networks like ad hoc networks [19], which do not depend on a central unit for data processing.

## 3. Framework Design

The framework design presented in this article has two important aspects. First, by using a heterogeneous system, it enables connecting new units, be them or not robotic agents. Second, it sets a decentralized ad hoc network for communications between agents, avoiding the need for permanent communications by creating a record history based on the last information transfer and the RSSI indicator. To achieve this, it is important to describe the required characteristics for agents in the MARS, along with a description of framework processes.

### 3.1. Agent Description of the MARS

**Figure 1:**
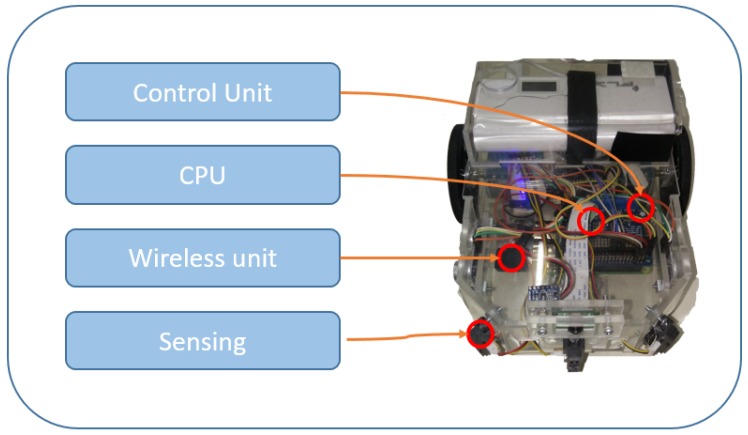
Robotic agent diagram.

**Figure 2:**
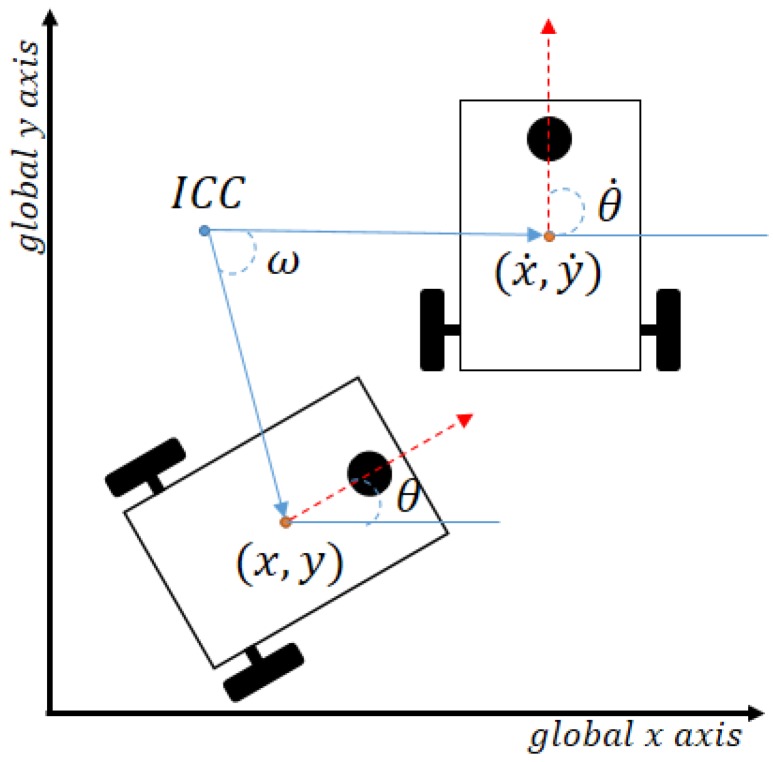
Dynamic model of a two-wheeled differential robot.

Since the proposed system is heterogeneous, two types of agents are possible, derived from the principle of WSN nodes. The first type of node is a robotic agent, which can be UAVs or UGVs. They need to include a sensor unit to capture information from the environment, a control unit for actuators to enable their movement or making changes in the environment, a unit to process the information captured or transmitted by another agent, and finally, a wireless unit that accepts ad hoc infrastructure, to send and receive information from other agents in the system, as shown in Figure 1. These agents also need to have a description of the dynamic model to represent their own behavior in the system. This allows the design of controllers for formation or localization [40]. Equation (1) represents the dynamic model of the differential non-holonomic robot used in the experiments in this article (see Figure 2). Non-holonomic agents cannot displace directly in the axes (x,y), and their spin and displacements are represented by Instantaneous Center of Curvature (ICC), where they depend on the radius R, and the position (x,y) depends on the robotic agent so that $ICC=\left[x-R\sin\theta ,y-R\cos\theta \right]$.

(1) $$\left[\begin{array}{c} \dot{x}\\ \dot{y}\\ \dot{\theta } \end{array}\right]=\left[\begin{array}{ccc} \cos\left(\omega \delta t\right) & -\sin\left(\omega \delta t\right) & 0\\ \sin\left(\omega \delta t\right) & \cos\left(\omega \delta t\right) & 0\\ 0 & 0 & 1 \end{array}\right]\left[\begin{array}{c} x-ICC_{x}\\ y-ICC_{y}\\ \theta \end{array}\right]+\left[\begin{array}{c} x\\ y\\ \omega \delta t \end{array}\right],$$

where:

$\dot{x}=$ New position *x*-axis,
$\dot{y}=$ New position *y*-axis,
$\dot{\theta }=$ New orientation,
θ= Angle of orientation,
ω= Angle respect the ICC.

**Figure 3:**
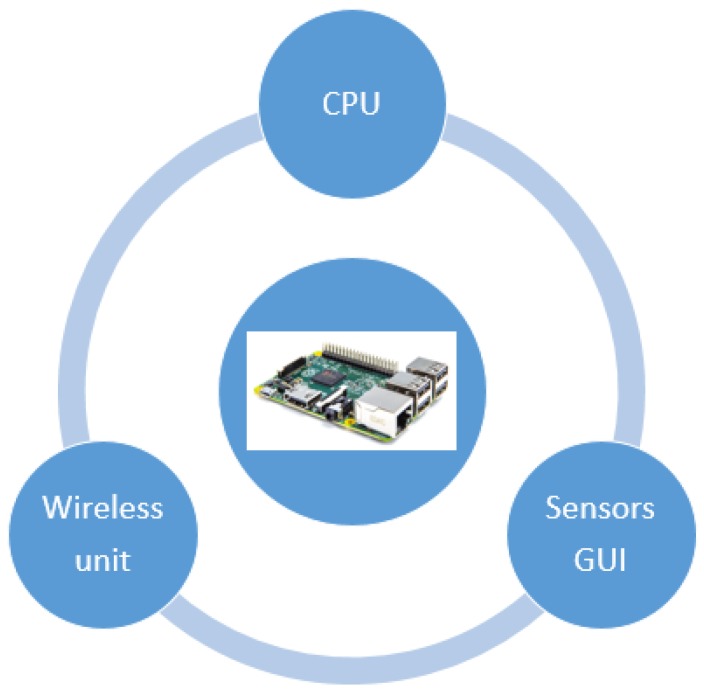
Monitor or sensing unit diagram.

The second type of agent describes monitoring units and wireless sensors, which are static agents characterized by having a wireless communication unit that accepts ad hoc infrastructure mode to establish the communication with the system, a processing unit to manipulate the acquire data, and either a sensor unit or a Graphical User Interface (GUI) to interact with the environment or a user, depending on if it is a WSN node or a monitor agent. The diagram for the monitor agent used in this article is shown in Figure 3.

It is important to highlight that the robot and monitor agents must have an Wi-Fi adapter that support an ad hoc infrastructure with the 802.11n protocol. In the case of the chips as the ESP8266 [41] that are cheap and have an embedded Wi-fi adapter, does not have the possibility to configure or connect an external unit to support an ad hoc infrastructure. The agents in Figure 2 and Figure 3 uses a Raspberry Pi 3, and this has embedded a BCM43143 [42] . A Wi-Fi chip adapter that does not support an ad hoc infrastructure. To solve this, it is mandatory to connect a wireless adapter as the TL-WN823N [43] that support the ad hoc infrastructure mode and can be used by the Raspberry Pi 3.

### 3.2. Framework Description

The framework presented in this article is a procedure for decentralized communication between agents for monitoring or navigation purposes. The framework has five modular processes that can be modified at any moment on a system or level agent. However, when the system is first started, they must be executed sequentially.

The first process is the characterization of the agents’ components and their relationship with the environment; the second is the analysis of transmissions between agents; the third is initializing agents for their connection to the ad hoc network; the fourth process is the generation of the activity history in each agent in the system. Finally, the fifth process describes information transmission through a header to be included in the information packet. These five processes are explained as follows.

#### 3.2.1. Characterization of System Agents

Each of the elements in the environment needs to be classified. This includes knowledge about the components of each agent, the environment, and establishing the initial location points for new agents if the environment is unknown or knowing the location of wireless nodes pre-installed in the area. To establish the initial location of mobile agents in an unknown environment with a surface area of $D=\left[D_{x},D_{y}\right]$, a number of agents $A_{n}$ is considered as the initial members of the MARS so that *n* is a numeric identifier; each agent with an odometer module capable of processing the traveled distance by pulses per rotation (*ppr*) and the radius *r* of its wheels, calculating the distance per pulse (dpp) through Equation (2):

(2) $$dpp_{n}=\frac{2\pi r_{n}}{ppr_{n}}.$$

**Figure 4:**
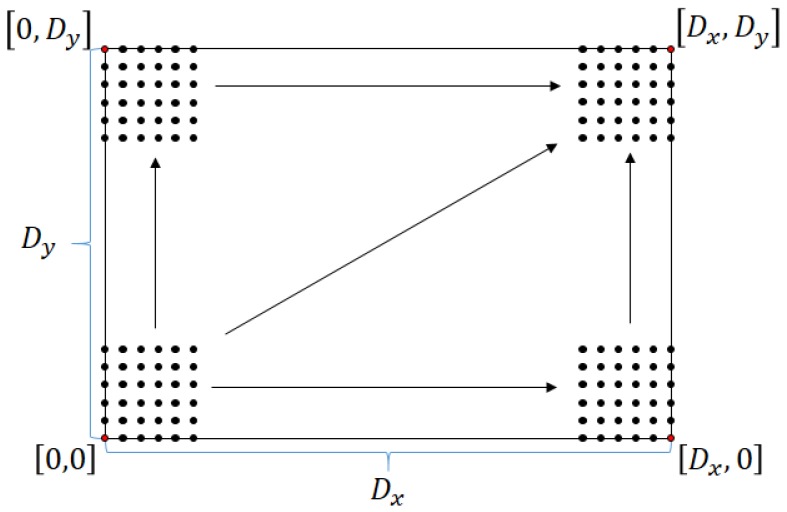
Odometry point generation.

To generate a dot matrix $p_{g}=x_{g}\times y_{g}$ of location in the environment, a lower value of $p_{g}$ equal to $\left[0,0\right]$ is selected and an upper value calculated by the Equation (3)—in this case, $\left[D_{x},D_{y}\right]$ is the environment area (see Figure 4). By generating this grid, it is possible to locate agents in known points, simplifying navigation tasks [19]:

(3) $$p_{g(sup)}=\left[\frac{D_{x}}{max\left(dpp_{n}\right)},\frac{D_{y}}{max\left(dpp_{n}\right)}\right].$$

#### 3.2.2. Data Transmission Analysis

Information exchange of MARS in real environments represents a challenge, where, to increase agent autonomy, it is indispensable to avoid information loss, increase fault tolerance, and reduce energy consumption [24,44,45]. The Point-to-Point (PTP) transmission model provides a high level of fault tolerance by not requiring maintaining communication with the whole system, which does not need a central unit reducing the bottlenecks in the communication system, which is ideal for MARS [46]. This model must be complemented by a communication protocol for information transfer. The two most common protocols used are User Datagram Protocol (UDP) and Transmission Control Protocol (TCP).

UDP sends information packets without needing to validate their correct reception, allowing for higher transference rates but at the same time increasing error rates. Alternatively, TCP prevents information loss by validating each packet sent between agents, but, in cases where agents have a weak link, this is reflected in a higher packet loss rate, increasing energy usage and time when there is an undefined confirmation loop between agents [47].

**Figure 5:**
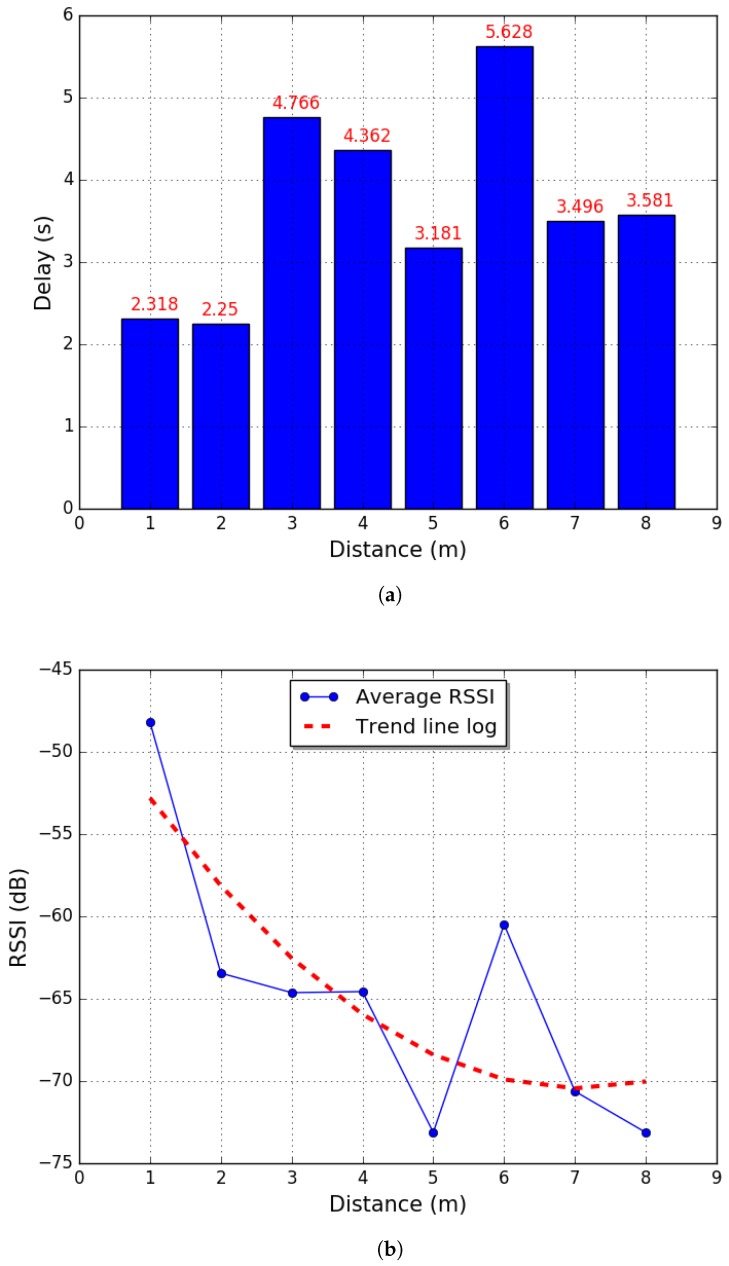
Wireless data transmission analysis (**a**) information transmission delay; (**b**) RSSI power level indicator.

To reduce energy usage, information transmission delay, and confirmation loop in TCP connections, this framework proposes a process to analyze the wireless modules used in MARS agents before deploying them in the environment. Figure 5 shows the characterization of wireless adapter TL-WN823N in an environment with a concrete wall as an obstacle. In this process, the source behind the wall is located at an initial distance from the wall of 1 m. The distance is subsequently increased in steps of 1 m, and, at each location change of the wireless nodes, the network is flooded with packets of different sizes. This allows for analyzing RSSI, the amount of data lost, and information transmission delay problems, where the last one can impact dramatically the behavior of the system [48]. This node separation is performed until there the data transmission shows information loss, which in this particular case was at 8 m.

#### 3.2.3. Agent Initialization

For agents to be able to enter the network, it is important that they comply with the OSI-based four-layer communication model. The first layer is the physical layer, where each agent in the heterogeneous system needs to have a wireless communication model. The second layer is the link layer, in this case, the 802.11n standard, which is in charge of initializing the client name in the wireless network. It is obligatory that all agents use the same name, as any agents that do not use the same name will not be able to share information in the network. The third layer is the network layer, and it uses the IP protocol. Every agent in charge of initializing agents being initialized into the network needs to assign them a unique address and be capable of adding new agents to the network by assigning them an address through the Dynamic Host Configuration Protocol (DHCP) . To avoid address collisions, Equation (4) is implemented in the assignment of addresses to initial agents and Equation (5) to assign their address range, where *A* is the number of addresses available, *N* is the number of initial agents in the system, and *i* is the agent indicator with values from 0 to N−1. The last and fourth layer is the transport layer, using the TCP protocol to ensure packet transmission without information loss:

(4) $$address_{\left(i=0\right)}=round\left(\frac{A+2}{N}i+2\right);address_{\left(i>0\right)}=round\left(\frac{A+2}{N}i\right),$$

(5) $$DHCP_{i}=\left[address_{i}+1\;..\;round\left(\left(\frac{A+2}{N}\left(i+1\right)\right)+1\right)\right].$$

#### 3.2.4. History Generation

The history of each of the agents in the system is represented by a dynamic array ($Ah_{xi}$) of four positions without including the index, user as a title in the array to identify the information transfer of the agent with the system, on Table 1. The first position in the history contains the ID of the agent that transferred the information, where the range is equivalent to the subnet mask selected in the link layer during agent initialization. The second position is the ID of the agent receiving the information, which is also in a range that depends on the subnet mask, as in the first position. The third position stores the packets pending transmission between both agents identified in the first and second position. Finally, the fourth position defines the packets pending transmission between both agents. The range of the last two positions will depend on the agent’s available memory for storing information. The update of the history between two agents (*A* and *B*) can occur according to the following three rules:

1. In agent *A*, the identifier on the array Ad is not contained in the first slot of the history Ah.
2. In agent *A*, the identifier on the array Ad is in the first slot, but the second slot does not contain the ID of agent *B*.
3. In agent *A*, the identifier on the array Ad is in the first slot, and the second slot contains the ID of agent *B*, but the fourth slot has a value greater than zero.

To create the history in an agent, the dynamic array with the four positions mentioned has to be created with an initial length of zero. Besides this, another empty unidimensional dynamic array must be initialized to store the IDs from which information has been received ($Ad_{xi}$). To understand how the information stored in the history is handled, we propose an environment with three robotic agents with the following IDs: $Ro_{1}$ (192.268.1.2), $Ro_{2}$ (192.268.1.85), and $Ro_{3}$ (192.268.1.170). Each of these agents has an array of stored data ($Ad_{xi}$), as shown in Table 2.

The three agents belong to the same network, with a subnet mask 255.255.255.0, and the last value of the IP address establishes the identifier for each agent. The first information transfer occurs between agents $Ro_{1}$ and $Ro_{2}$. As this is the first time information will be transferred between these two agents, the first rule for history update applies (see Table 3).

The second transfer occurs between agents $Ro_{2}$ and $Ro_{3}$. In this case, agents $Ro_{2}$ and $Ro_{3}$ fulfill the first rule for history updates, but, additionally, agent $Ro_{2}$ fulfills the second rule because it has already transferred information to agent $Ro_{1}$ (see Table 4). Finally, as agent $Ro_{2}$ has new information, it will update its history when it sends the new information to agent $Ro_{1}$ (Table 5).

#### 3.2.5. Data Package Header

The header for packet transmissions has three parts. The first part is code, the second is the value requested by the code (optional), and the third part is the information requested by the agent. The code is the identifier that corresponds to the type of transmission that will occur between the robotic agents. This code can have a value between **0** and **3**, where code **0** is used to query the number of packets pending in a robotic agent, code **1** is used to request a determined number of packets, code **2** is used to begin packet transmission, and code **3** is used to transfer a packet immediately.

**Figure 6:**
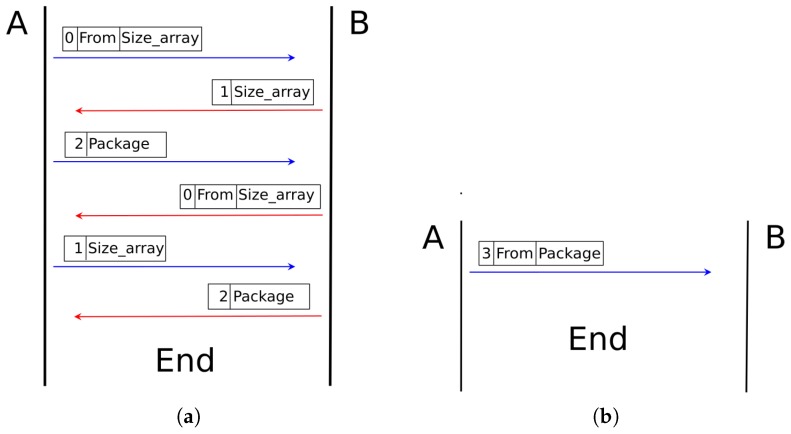
Wireless data transmission analysis. (**a**)data transfer through an information request , (**b**)data transfer through an immediately request.

According to the code included at the beginning of the packet header, the data transfer protocol can generate an information request or an immediate delivery. Figure 6a shows a data transfer through an information request between two agents. Agent *A* sends agent *B* the code **0**, followed by the data source ID, and finally the amount of data it has about the source to agent *B*. Then, agent *B* replies to agent *A* with code **1** and the amount of data it requires from the source previously sent to agent *A*. Finally, agent *A* sends code **2**, so it can then send the data packet requested. This will only happen if and only if the agent *A* history includes information from agent *B*. If it does not, the transmission will occur immediately, as shown in Figure 6b. In that case, code **2** is used, followed by the complete packet from agent *A* to *B*.

The information transfer between two agents will occur when they are within the configured RSSI range, and, at the time, one of them has new information in its history in the column that stores data pending to be sent. The latter condition will be fulfilled when the agent captures new data from the environment or when it has received information from another agent. In this case, the data transfer by information request will be used. In any other case, if the source agent does not have in its history information to be transmitted to another agent and is within range, it will send immediately all the information packet stored. This process is shown in Algorithm 1.

**Algorithm 1** Data transfer between agents.
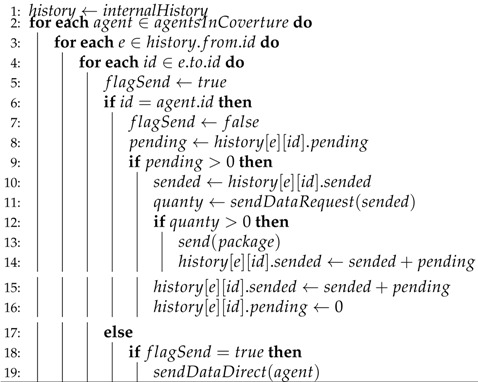


## 4. Decentralized Framework in MARS

To create the non-convex environment and the dynamic model of the robotic agents, we used the V-Rep simulator, with the goal of validation the decentralized framework [49]. This simulator allows the creation of obstacles and robotic agents, which can be controlled directly from Python and wireless adapters can be created from the Sockets library [50]. The environment is configured in an area of 25 m${}^{2}$, generating in its interior area a maze with cells separated by a minimum of 50 cm that allows enough space for mobile robots to navigate.

Robotic agents in this environment need to be capable of detecting the walls and tracing topographic maps independently. These must be shared with all the agents in the system, guaranteeing that each agent will contain the complete topological map of the environment through the use of this framework.

Initially, the system will be comprised of two mobile robotic agents. Another mobile robotic agent will be subsequently added to the system. In a final stage, a monitor agent will be added to the system to validate the heterogeneousness of the framework to accept different types of agents in real time.

### 4.1. Experiment with Two Robotic Agents in the System

**Figure 7:**
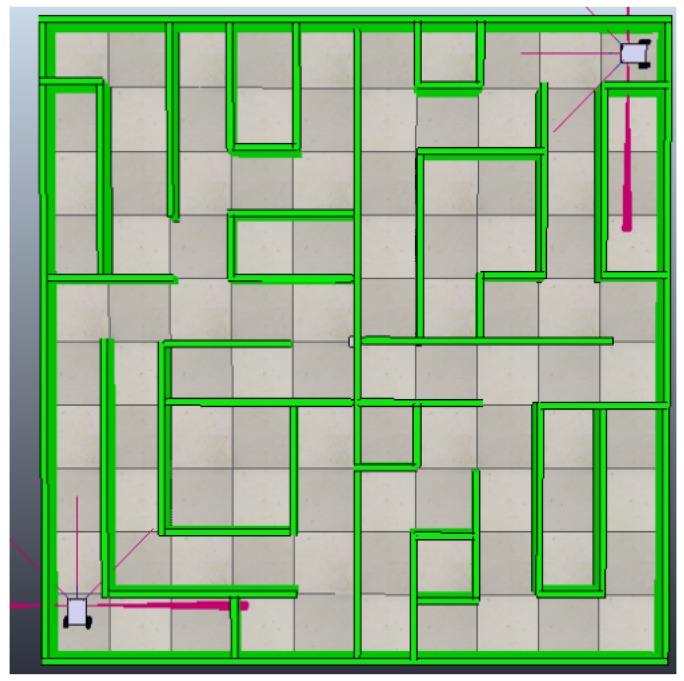
Experiment with two robotic agents.

As this is the first time that the agents will be inserted in the environment, they must follow the five steps of the framework in an organized way. The first process is the characterization of the agents in the system. Each simulated robotic agent is constructed under the physical model shown in Figure 1 and is placed in an opposite end of the environment in such a way that they follow an independent path. These agents are equipped with encoders that allow to read odometry directly from the motor axis and independently. By using Equation (2), a value of dpp = 0.7853 mm is obtained, generating a dot matrix in the environment through Equation (3). This locates $robot_{1}$ at the position (0.25 m, 0.25 m) or in the matrix pg(318,318). The second robotic agent is located at the position (4.75 m, 4.75 m) or in the matrix pg(6048,6048), as shown in Figure 7.

The second process is the analysis of information transmission. As the agents are equipped with a Wi-Fi adapter TL-WN823N (TP-Link, Shenzhen, China), a maximum transmission distance of 2 m is selected. This is when the network is recognized with an RSSI greater than −63.4375 dB. In this case, each data stored in the dynamic array will include one byte to store the vertices of the graph with two 16−bit floats to represent the position of vertices (x,y) of the topological map created by the robotic agents.

The third process is agent initialization, with an access mask of 255.255.255.0 and a gateway with the IP address 192.168.1.1, which allows assigning 253 agents to the system. IP assignment uses Equations (4) and (5), where the agent $robot_{1}$ is assigned an IP address 192.168.1.2 from a DHCP address pool that goes from 192.168.1.3 to 192.168.1.127. Agent $robot_{2}$ is assigned an IP address 192.168.1.128 from a DHCP address pool that goes from 192.168.1.9 to 192.168.1.254.

The fourth process is assigning the history to each agent. This process consists of creating a dynamic array of $\left[x\right]\times \left[4\right]$ positions, where *x* is the dynamic index that will increment after each transmission or reception of information from other agent in the environment. The last process is performed in the application layer of the agent.

Once the five processes have been completed in the environment, the system starts the implementation of the task, which is the generation of a topological map of the environment, in which the vertices are shared by the communication model of the fifth process of the framework.

**Figure 8:**
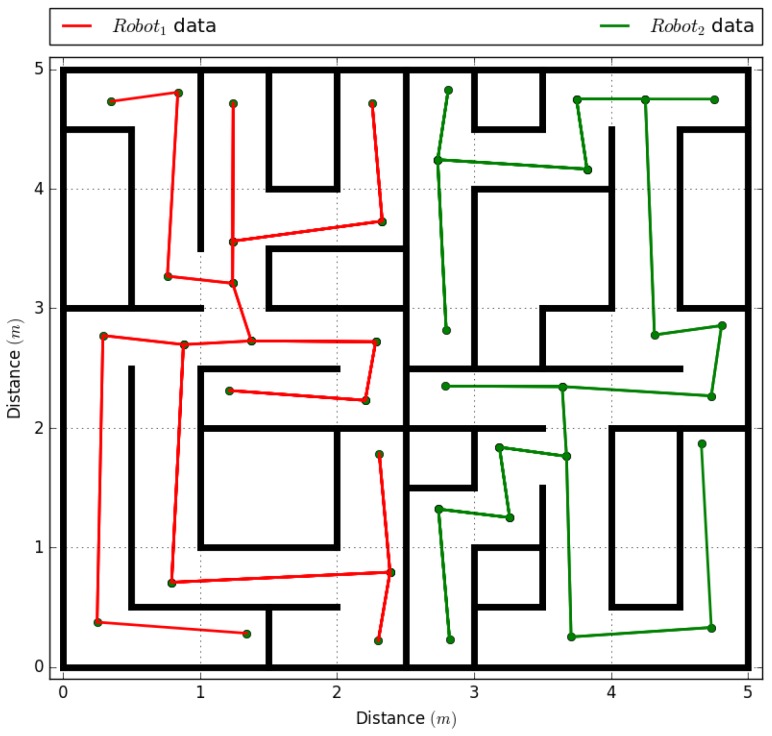
Topological map processed by two robotic agents.

The experiment is performed by comparing a centralized system and a decentralized system and measuring the distance, time used, and the number of bytes sent to obtain the complete topological map of the environment (Figure 8). The results of both experiments can be seen in Table 6. A centralized system using the communications model of this framework shows a 15.87% reduction in the time to send the map to the agents, compared to the decentralized system. However, the decentralized system using full use of the framework shows a 50% reduction of data traffic to generate the map, reducing energy use of robotic agents [51].

### 4.2. Experiment with Three Robotic Agents in the System

**Figure 9:**
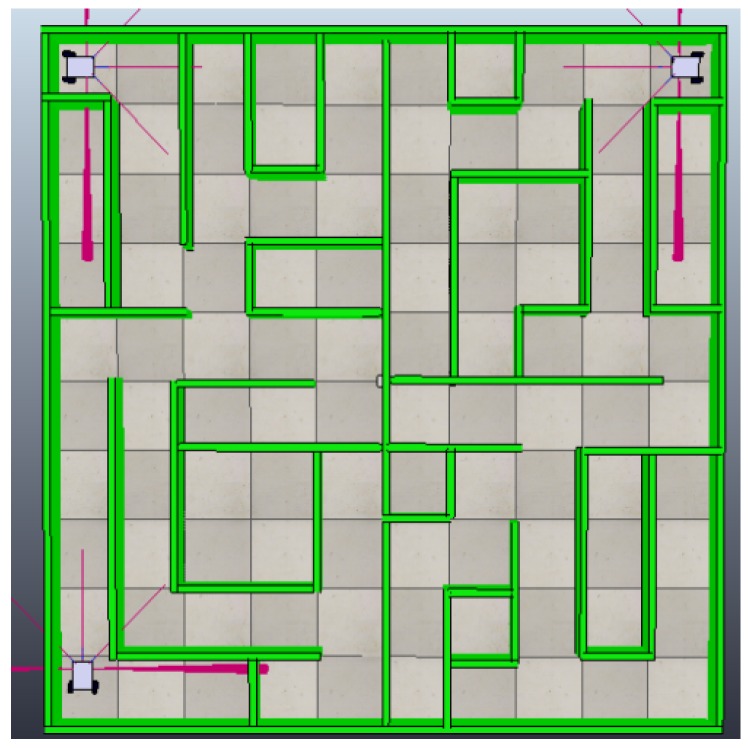
Experiment with three robotic agents.

By characterizing the robotic agents and their transmission, we continue with the implementations of processes 3 and 4 of the proposed framework, taking into account that the new robotic agent will be located at the position (0.25 m, 4.75 m) or in the matrix pg(318,6048), and the two existing agents will be located in their initial positions, as shown in Figure 9. By adding a new agent to the system, we continue with process 3 to initialize the agents, again using Equations (4) and (5). Agent $robot_{1}$ is assigned an IP address 192.168.1.2 from a DHCP address pool that goes from 192.168.1.3 to 192.168.1.84. Agent $robot_{2}$ is assigned an IP address 192.168.1.85 from a DHCP address pool that goes from 192.168.1.86 to 192.168.1.169. Finally, agent $robot_{3}$ is assigned an IP address 192.168.1.170 from a DHCP address pool that goes from 192.168.1.171 to 192.168.1.254.

**Figure 10:**
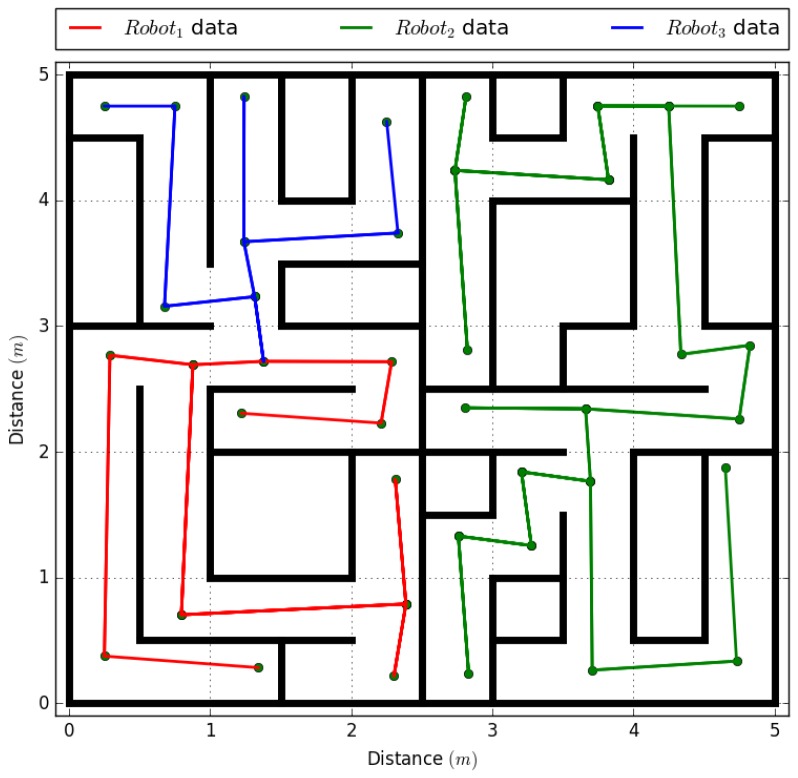
Topological map processed by three robotic agents.

The fourth process only adds the dynamic array of $\left[x\right]\times \left[4\right]$ positions to agent $robot_{3}$, and agents $robot_{1}$ and $robot_{2}$ have their histories created in the previous experiment erased. Again, by doing these processes, the continue with the generation of the topological map, as shown in Figure 10, comparing distance, time, and the quantity of bytes sent to obtain the full topological map of the environment, both in a centralized and a decentralized system. The results of both experiments can be seen on Table 7. As in the experiment with two robotic agents, the centralized system shows a time reduction of 14.55% in information transmission to the complete system, while the decentralized system reduced the quantity of bytes sent in the system.

**Figure 11:**
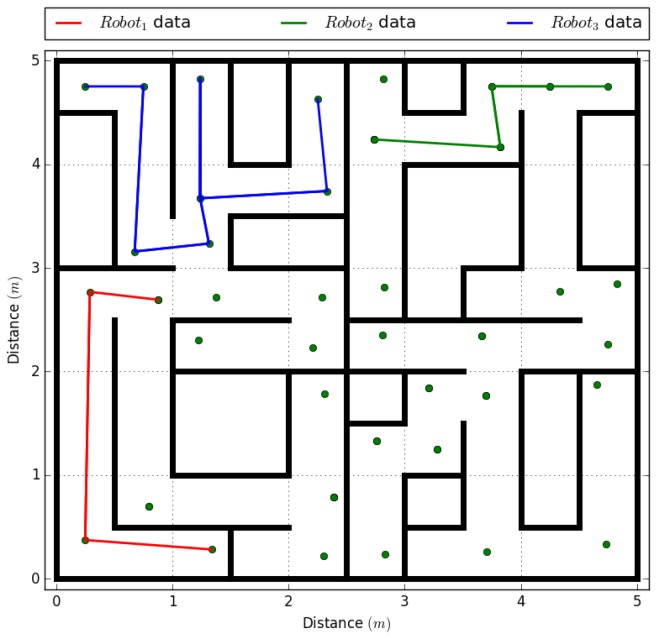
Topological map processed by the $robot_{3}$ agent after its failure, with the information sent from the $robot_{1}$ and $robot_{2}$ agents.

The experiment was repeated but making the central system fail in the centralized system, and, in the decentralized system, the agent $robot_{3}$ was caused to fail, as shown in Table 8. In the first test, the system could not complete the full map because it did not have a unit that received the information and relayed it to the rest of the agents, so each agent obtained a partial map of the environment. In the decentralized test, although the agent $robot_{3}$ failed at the 110 s mark after the test start, the remaining agents were able to complete the topological map of the environment. The results taken from the agent that failed are shown in Figure 11, where the points without a connection are the missing vertices.

**Figure 12:**
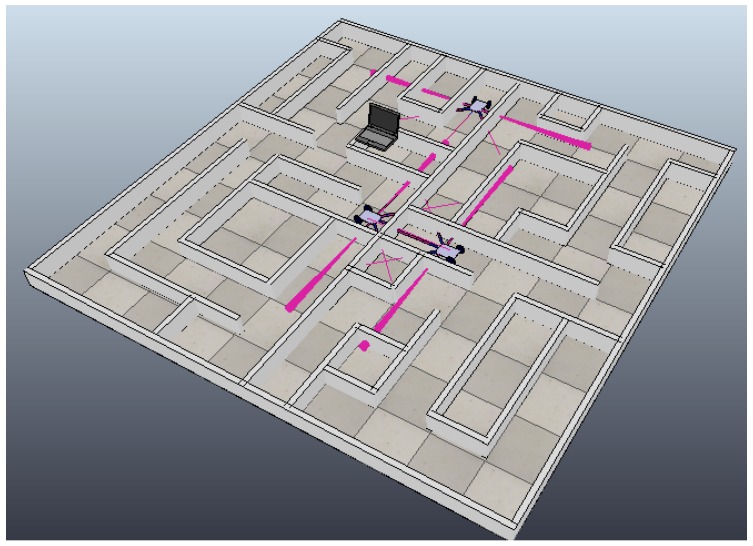
Experiment with three robotic agents and a monitor agent.

Additionally, to overcome the mechanical failure of $robot_{3}$, a monitoring agent was added to seize the capacity of the system to be heterogeneous. Monitoring agents need to comply with the characteristics shown in Section 3.1. This monitoring agent was implemented with the same model of wireless adapter used in the rest of the robotic agents, so only the fourth process of the framework was applied to be able to establish communication with the agents already deployed. The monitoring agent $monitor_{1}$ is placed at the coordinates (1.75 m, 3.25 m) as shown in Figure 12, which allows it to be within range of agents $robot_{3}$ and $robot_{1}$. As it had better coverage with agent $robot_{1}$, the monitoring agent connects directly with $robot_{1}$ and is assigned the IP address 192.168.1.3. Once the IP address was assigned, the process of communication and data transfer from $robot_{1}$ to $monitor_{1}$ is initiated. After this, $monitor_{1}$ sends the data to the agent $robot_{3}$. The quantity of bytes transmitted in this new transfer is shown in Table 9.

## 5. Conclusions

MARS decentralization has become a very important area of cooperative robotics. By not depending on a central unit for processing or traffic control between agents, it allows increasing the autonomy and fault tolerance of the whole system. The biggest contribution of this article is the development of a framework for communications between multiple agents in a decentralized system.

The difference with the existing communication models is that this framework does not depend on a central unit for data processing or collection. It also does not require permanent communications between agents in the system, allowing for real-time connection of different agents, robotic or monitoring, at any moment, as long as they are within the number of assignable addresses defined in this framework’s third initialization process.

The results of different simulations in a non-convex environment are shown, assessing the communication in the system in the creation of a topological map of the environment. The results show the framework’s improved performance compared to traditional centralized systems, in the reduction of the amount of data transmitted between agents, the fault tolerance in the case of failure of an agent, and the capacity to accept new agents in the system. However, centralized systems require less time to share the information with the whole system.

Although none of the agents modeled in the system had a module for SLAM tasks, it was possible to generate a topological map through processing odometry data and obstacle assessment through distance sensors.

Future works will use this framework to evaluate its functionality in MARSs in emergency response situations, specifically in search and rescue operations of people, using different metrics to facilitate the detection of malicious or problematic agents to improve the performance of the network as is shown by Gutiérrez et al. [52]. Additionally, as this is a modular framework, its implementation in a sensor network will be studied, incrementing the number of agents that can participate in the system, for data analysis purposes in different environments to control the temperature, humidity and water level in crops as is shown in Anzola et al. [53].

We are also researching the process for integrating this framework in real-time vehicular traffic analysis, with consideration that not only will the data be distributed within the system, but also will need to be sent to a remote database through an Internet of Things (IoT) concept, as shown in Jiménez et al. [54]. Furthermore, we are studying the possibility of integrating other rules into our framework to recover the communication when an agent suffers a failure, as the work presented by Shang [55].

## Figures and Tables

**Table 1:** Dynamic array for the Data and the History.

Array-Data			Array-History				
Index	ID	Data	Index	From	To	Send	Pending

**Table 2:** History initialization with three agents.

192.168.1.2								192.168.1.85								192.168.1.170							
Ad			Ah					Ad			Ah					Ad			Ah				
Index	ID	D	Index	F	T	S	P	Index	ID	D	Index	F	T	S	P	Index	ID	D	Index	F	T	S	P
0	1	x0						0	2	y0						0	3	z0					
	1	x1							2	y1							3	z1					
	1	x2															3	z2					
																	3	z3					

**Table 3:** Data transference between $Ro_{1}$ and $Ro_{2}$.

192.168.1.2								192.168.1.85							
Ad			Ah					Ad			Ah				
Index	ID	D	Index	F	T	S	P	Index	ID	D	Index	F	T	S	P
0	1	x0	0	1	2	3	0	0	2	y0	0	2	1	2	0
	1	x1	1	2	1	2	0		2	y1	1	1	2	3	0
	1	x2						1	1	x0					
1	2	y0							1	x1					
	2	y1							1	x2					

**Table 4:** Data transference between $Ro_{2}$ and $Ro_{3}$.

192.168.1.85								192.168.1.170							
Ad			Ah					Ad			Ah				
Index	ID	D	Index	F	T	S	P	Index	ID	D	Index	F	T	S	P
0	2	y0	0	2	1	2	0	0	3	z0	0	3	2	4	0
	2	y1	1	1	2	3	0		3	z1	1	2	3	2	0
1	1	x0	2	1	3	3	0		3	z2	2	1	3	3	0
	1	x1	3	2	3	2	0		3	z3					
	1	x2	4	3	2	4	0	1	2	y0					
2	3	z0							2	y1					
	3	z1						2	1	x0					
	3	z2							1	x1					
	3	z3							1	x2					

**Table 5:** Data transfer between $Ro_{2}$ and $Ro_{1}$, sending data from $Ro_{3}$.

192.168.1.85								192.168.1.2							
Ad			Ah					Ad			Ah				
Index	ID	D	Index	F	T	S	P	Index	ID	D	Index	F	T	S	P
0	2	y0	0	2	1	2	0	0	1	x0	0	1	2	3	0
	2	y1	1	1	2	3	0		1	x1	1	2	1	2	0
1	1	x0	2	1	3	3	0		1	x2	2	3	1	4	0
	1	x1	3	2	3	2	0	1	2	y0					
	1	x2	4	3	2	4	0		2	y1					
2	3	z0	5	3	1	4	0	2	3	z0					
	3	z1							3	z1					
	3	z2							3	z2					
	3	z3							3	z3					

**Table 6:** Results from the experiment with two robotic agents.

Type	Robot IP	Distance (m)	Time (s)	Bytes Send
Centralized	192.168.1.2	30.5	472.46	60
	192.168.1.128	23.84	457.29	60
	192.168.1.129	–	–	120
Decentralized	192.168.1.2	34.90	561.58	60
	192.168.1.128	28.97	535.97	60

**Table 7:** Results from the experiment with three robotic agents.

Type	Robot IP	Distance (m)	Time (s)	Bytes Send
Centralized	192.168.1.2	18.63	289.09	36
	192.168.1.85	23.85	447.69	60
	192.168.1.170	7.94	133.56	24
	192.168.1.86	–	–	240
Decentralized	192.168.1.2	18.63	297.14	132
	192.168.1.85	29.20	523.93	75
	192.168.1.170	9.63	169.96	75

**Table 8:** Results from the experiment with three robotic agents after the failure of the $robot_{3}$ agent.

Type	Robot IP	Distance (m)	Time (s)	Bytes Send
Centralized	192.168.1.2	30.5	472.46	–
	192.168.1.85	23.85	447.69	–
	192.168.1.170	33.3	484.35	–
	192.168.1.86	–	–	–
Decentralized	192.168.1.2	18.63	297.14	48
	192.168.1.85	29.20	523.93	81
	192.168.1.170	6.67	109.70	69

**Table 9:** Experiment with three robotic agents and one monitor agent.

Agent	Robot IP	Bytes Send
$Robot_{1}$	192.168.1.85	168
$Robot_{2}$	192.168.1.85	81
$Robot_{3}$	192.168.1.170	69
$Monitor_{1}$	192.168.1.3	96

## Abbreviations

The following abbreviations are used in this manuscript:

MARS	Multi-Agent Robotic System
RSSI	Received Signal Strength Indication
SLAM	Simultaneous Localization and Mapping
WSN	Wireless Sensor Network

References
